# Association between a body shape index and cognitive impairment among US older adults from a cross-sectional survey of the NHANES 2011–2014

**DOI:** 10.1186/s12944-024-02165-2

**Published:** 2024-06-05

**Authors:** Yanwei Zhang, Peng Zhang, Dekun Yin

**Affiliations:** 1 Department of Anesthesiology, Shanghai Ninth People’s Hospital, Shanghai Jiao Tong University School of Medicine, Shanghai, China; 2 Department of Anesthesiology, Funing People’s Hospital of Jiangsu, Yancheng, Jiangsu province China

**Keywords:** A body shape index, Cognitive function, Cross-sectional survey, Older adults, NHANES

## Abstract

**Purpose:**

This study aimed to assess the relationship between A Body Shape Index (ABSI) and cognitive impairment among older adults in the United States.

**Methods:**

This cross-sectional study analyzed cognitive function in 2,752 individuals aged 60 and older using data from the 2011–2014 National Health and Nutrition Examination Survey (NHANES). Cognitive assessments were conducted using the Immediate Recall Test (IRT), Delayed Recall Test (DRT), Animal Fluency Test (AFT), and Digit Symbol Substitution Test (DSST). A Body Shape Index (ABSI) was calculated from waist circumference (WC), weight, and height. The relationship between ABSI and cognitive outcomes was examined through multifactorial linear regression, smooth curve fitting, and subgroup and interaction analyses.

**Results:**

With complete data, 2752 persons 60 and older participated in the study. After adjusting for covariables, these results showed statistically significant negative relationships between ABSI, IRT, and DSST scores. The negative correlation between DSST and ABSI is more substantial in males than females. There is less of a negative link between ABSI, AFT, and DSST among drinkers who consume 12 or more drinks annually compared to those who consume less. Furthermore, compared to individuals without high blood pressure(HBP), those who suffered HBP showed a more significant negative connection between ABSI and AFT.

**Conclusion:**

Lower cognitive function was linked to higher ABSI.

## Introduction

Cognition encompasses the mental activities of gaining knowledge and comprehension, such as thinking, experiencing, and utilizing the senses. It includes a range of functions such as language production, decision-making, attention, comprehension, memory and working memory, perception, knowledge formation, problem-solving, evaluation, calculation, and reasoning [1]. These cognitive processes are essential for efficiently navigating daily life and maintaining a high quality of life. Cognitive impairment involves a marked deterioration in one or more mental capabilities, going beyond the usual changes seen in normal aging. In older adults, this often manifests as dementia, predominantly caused by Alzheimer’s disease. The progression from normal aging to dementia typically begins with milder forms of cognitive decline, known as Mild Cognitive Impairment (MCI). Although Mild Cognitive Impairment (MCI) does not substantially affect daily activities, it signals an increased likelihood of developing Alzheimer’s disease [2, 3]. Recent research shows that about 60–80% of dementia cases in older adults stem from Alzheimer’s disease [4], emphasizing the critical need for timely intervention driven by associated high treatment costs, healthcare burdens, and productivity losses [5–7].

Recent data from NHANES indicate that between 1999 and 2020, the rate of obesity in the US increased from 30.5–42.4% [8]. Obesity is associated with a heightened risk of cardiovascular diseases and can adversely affect cognitive function and the central nervous system (CNS), posing both local and global challenges [9]. Two commonly used metrics to assess obesity are Body Mass Index (BMI) and waist circumference (WC); however, they do not distinguish between fat and muscle tissue [10]. . A newer metric, ABSI, demonstrates a strong correlation with mortality risk and excels in identifying visceral rather than peripheral fat, distinguished by its independence from BMI [11].

Recent studies have built a link between general obesity and cognitive impairment [12], yet detailed studies investigating the relationship between ABSI and cognitive function are scarce. This study posits that a higher ABSI, reflecting central obesity, correlates with reduced cognitive function in older adults. Utilizing a dataset from the NHANES 2011–2014, this research aims to address this gap by conducting an in-depth analysis of this relationship within a representative cohort of the US older adult population. The innovative aspect of this study is its emphasis on ABSI, an emerging metric that more accurately quantifies visceral fat and its potential effects on cognitive health, offering an advantage over traditional measures like BMI and waist circumference.

## Methods

### Study population

**Fig. 1:**
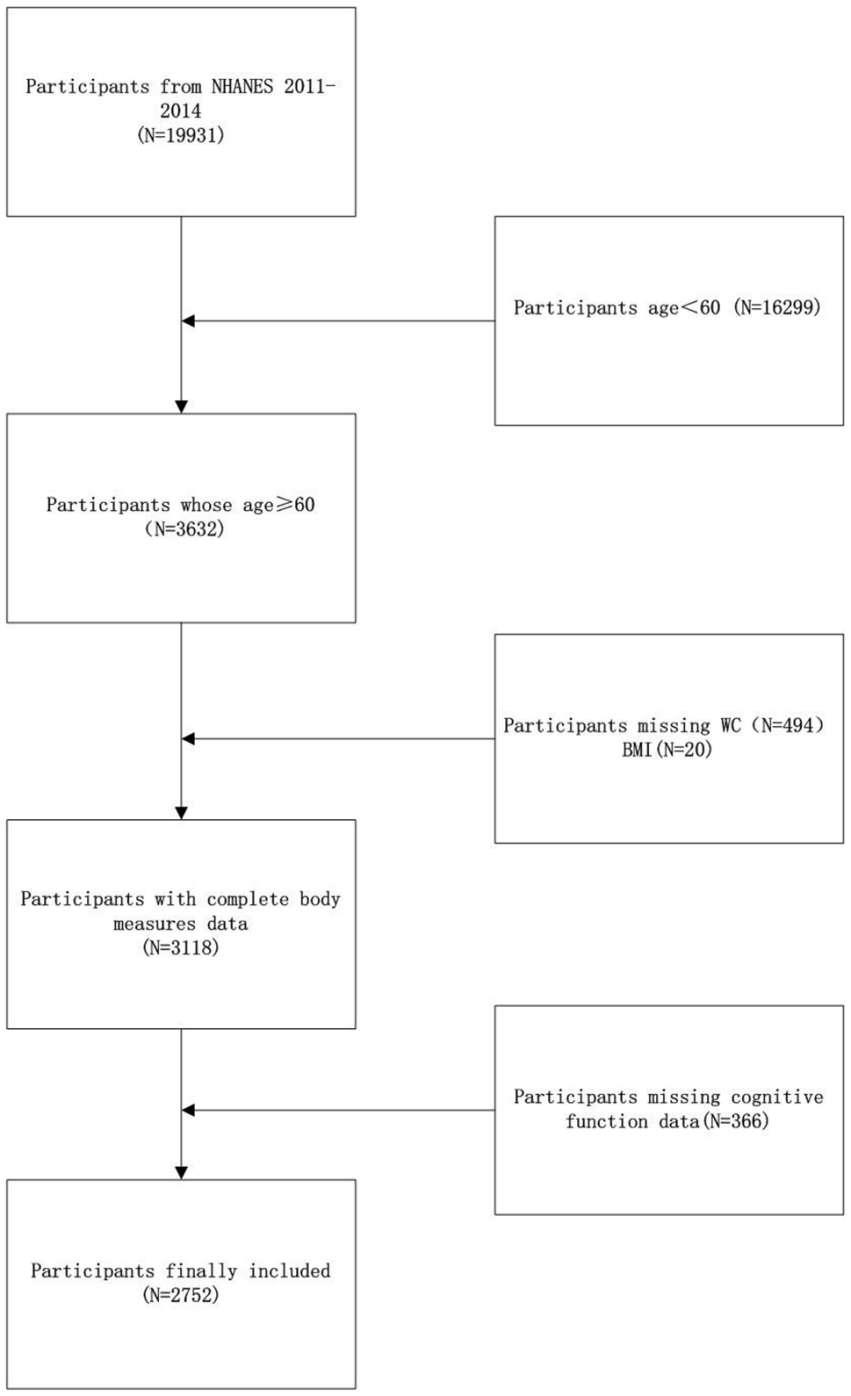
Flow diagram showing the participants’ selecting step

NHANES provides critical data to the public, researchers, and policymakers, facilitating the understanding and decision-making processes concerning nutrition and health. This study focuses on a dataset from 2011 to 2014, adhering to ethical standards by obtaining written consent. Figure 1 shows after excluding individuals younger than 60 years old(16,299), lacking waist circumference data (494), BMI data (20), and cognitive function data (366), the initial enrollment of 19,931 participants was reduced to 2,752 eligible subjects for the last step of the analysis.

### ABSI

ABSI, designed as a dimensionless index, signifies its independence from specific measurement units. The actual numerical value of ABSI does not directly quantify the degree of obesity but is utilized in statistical analyses to compare the relative risk of abdominal obesity across different individuals or populations, which is calculated as

$$ABSI = WC / \left({BMI}^{2/3} * {Height}^{1/2}\right)$$

### Measurement of cognitive function

Neuropsychological evaluations utilize tests such as the Animal Fluency Test (AFT), Digit Symbol Substitution Test (DSST), Immediate Recall Test (IRT), and Delayed Recall Test (DRT) to assess cognitive functions. This study applied the Consortium to Establish a Registry for Alzheimer’s Disease Word Learning (CERAD W-L), which includes the IRT and DRT components. These tests are crucial for evaluating memory skills and aiding in diagnosing cognitive impairments. Specifically, the CERAD W-L presents participants with ten unrelated words, requiring them to recall these words immediately over three trials. A delayed recall phase follows, which is initiated after a brief distractive task to evaluate both instant and delayed memory retention. To assess executive functions and verbal fluency, participants in the AFT test are required to speak the names of animals as much as possible within one minute. The AFT assesses vocabulary retrieval speed and dexterity, commonly indicating impairment in the prefrontal lobe. The DSST concentrates on attention, processing speed, working memory, learning, and hand-eye coordination, requiring participants to match symbols with numbers within two minutes. These tests are essential for diagnosing conditions such as Alzheimer’s disease, MCI, and other cognitive disorders, offering critical insights that guide the development of effective treatment and intervention programs.

Due to the lack of universal recognition of cognitive performance test cutoffs, this study adopted the twenty-fifth percentile of DSST scores as the threshold for defining low cognitive performance, aligning with established practices in existing literature [13]. Age-specific cutoffs were determined as follows: below 38 for ages 60 to less than 70, below 34 for ages 70 to less than 80, and below 29 for ages 80 and older, which accurately reflect the expected cognitive performance variation across these age groups. Participants scoring below these thresholds were classified into the Low Cognitive Performance Group, indicating potential cognitive impairment, while those scoring above were classified as having normal cognitive function, providing a robust framework for assessing age-related cognitive decline and facilitating targeted research into cognitive health interventions.

### Covariates

The analysis accounted for all relevant covariates: gender, age, educational level, race, income-to-poverty ratio (PIR), alcohol intake frequency, smoking history, diabetes, and high blood pressure (HBP). These factors were thoroughly incorporated to enhance the robustness of the findings and help control for confounding variables that might influence the relationship between ABSI and cognitive function. The NHANES database provides the research’s data and methodology for public use [14].

### ROC curve analysis for cognitive impairment prediction

This study took the Receiver Operating Characteristic (ROC) curve analysis to assess the discriminative capabilities of BMI, WC, and ABSI in predicting low cognitive function. Each metric was independently used in separate ROC analyses to determine their respective Area Under the Curve (AUC) values. This method allowed us to evaluate and compare the effectiveness of each metric in distinguishing between participants with normal and impaired cognitive functions. This approach enabled a direct comparison of how well each anthropometric index predicts cognitive impairment, aiding in identifying the most effective metric for the study group.

### Statistical analysis

This research conducted statistical analysis using EmpowerStats (version 4.2) and R software (version 4.2). Participants were divided into ABSI tertiles, and both chi-square and t-tests were taken to evaluate demographic disparities. Multivariate linear regression models were applied to explore the associations between ABSI and cognitive impairment. A trend test was conducted to assess the linear progression of cognitive function across ABSI tertiles. To visually represent the relationship between cognitive performance and ABSI, smooth curve fitting techniques were utilized. After categorizing DSST scores, the effects of ABSI, BMI, and WC on cognitive function were compared using Receiver Operating Characteristic curves (ROC). Interaction tests and subgroup analyses were also conducted to pinpoint correlations within specific demographic groups. A two-tailed p-value of less than 0.05 was considered statistically significant.

## Results

### Baseline characteristics

Table 1 illustrates the demographic characteristics of the study groups, stratified into tertiles according to ABSI. This study included 2,752 individuals aged 60 years and older, with a nearly balanced gender distribution of 51.02% females and 48.98% males. The mean ABSI was 0.084 ± 0.005, and the average age was 69.25 ± 6.73 years. ABSI tertiles were divided into lower (T1: 0.065–0.082), middle (T2: 0.082–0.086), and upper (T3: 0.086–0.108) ranges. The high ABSI subgroup differed from the low ABSI subgroup in various aspects, such as higher mean age, a greater proportion of men, lower PIR, reduced education levels, higher rates of cigarette taking, frequent alcohol intake, and prevalence of HBP and diabetes. In all tertiles, non-Hispanic whites predominated regarding racial distribution, especially in the middle and upper ABSI ranges.

**Table 1:** Basic characteristics of participants by ABSI tertiles among US older adults

Characteristics	Adjusted body size index			*P* value
	T1(0.065 ~ 0.082)	T2(0.082 ~ 0.086)	T3(0.086 ~ 0.108)	
Age(years)	67.51 ± 6.21	68.60 ± 6.54	70.90 ± 6.59	< 0.0001
Sex Female	72.97	46.76	43.47	< 0.0001
PIR	3.23 ± 1.51	3.17 ± 1.55	2.92 ± 1.52	< 0.0001
BMI (kg/m^2^)	29.94 ± 7.18	28.99 ± 5.77	28.03 ± 5.22	< 0.0001
Waist(cm)	97.56 ± 14.81	102.89 ± 14.24	106.53 ± 13.67	< 0.0001
Race (%)				< 0.0001
Mexican American	3.33	4.12	3.03	
Other Hispanic	3.83	4.55	2.96	
Non-Hispanic White	77.25	78.3	82.26	
Non-Hispanic Black	11.63	8.25	5.09	
Non-Hispanic Asian	3.5	3.86	2.86	
Other Race	0.47	0.92	3.81	
Education (%)				0.002
< high school	4.02	7.14	5.36	
9-11th grade	9.24	7.84	13.25	
High school graduate	22.12	21.82	21.84	
Some college or AA degree	31.9	33.78	29.74	
College graduate or above	32.73	29.41	29.76	
Don’t Know			0.05	
Smoked ≥ 100 cigarettes Yes (%)	42.46	54.22	55.11	< 0.0001
Alcohol intakes ≥ 12drinks/year Yes (%)	69.82	76.27	74.15	0.007
High blood pressure Yes (%)	51.72	58.91	63.57	< 0.0001
Diabetes (%)				< 0.0001
Yes	13.32	17.9	25.71	
No	84.03	77.02	70.01	
Broadline	2.65	5.08	4.28	
IRT	20.82 ± 4.35	19.61 ± 4.44	19.11 ± 4.33	< 0.0001
DRT	6.65 ± 2.20	6.30 ± 2.12	5.97 ± 2.38	< 0.0001
AFT	18.79 ± 5.62	18.33 ± 5.53	17.73 ± 5.77	0.0003
DSST	56.99 ± 16.66	51.75 ± 15.79	49.47 ± 16.04	< 0.0001

Mean ± SD for continuous variables: the P value was calculated by the weighted linear regression model; (%) for categorical variables: the P value was calculated by the weighted chi-square test

*Abbreviations* ABSI A Body Shape Index, IRT Immediate Recall test, DRT Delayed Recall test, AFT Animal Fluency Test, DSST Digit Symbol Substitution Test, WC Waist Circumference, BMI Body Mass Index, PIR Poverty Income Ratio, HBP High Blood Pressure

### Relationship between ABSI and cognitive performance

**Fig. 2:**
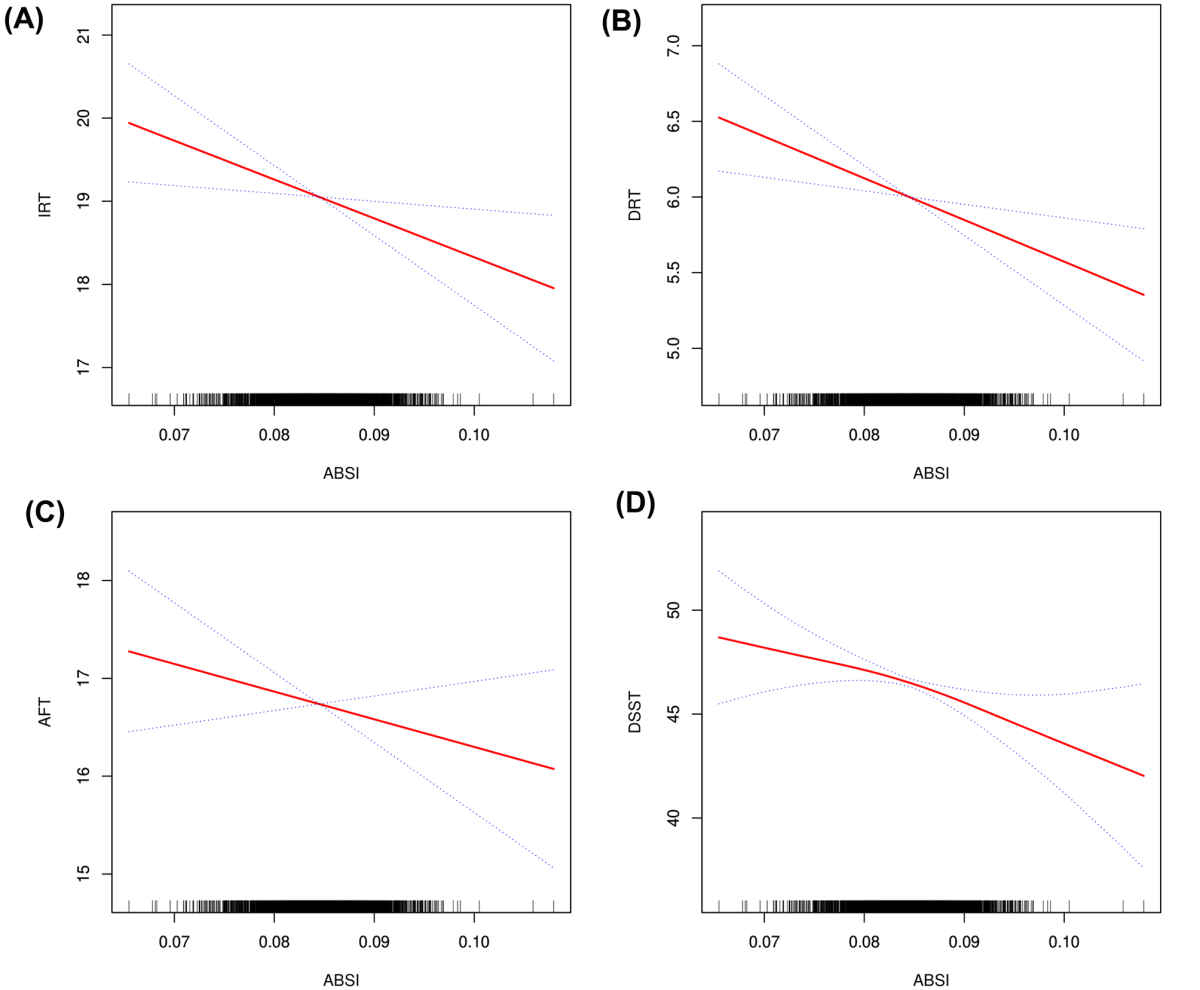
The nonlinear negative relationship between ABSI and cognitive function. Legend Solid red lines represent the smooth curve fit between the variables, while blue bands indicate the 95% confidence interval derived from this fit. (**A**) ABSI and IRT; (**B**) ABSI and DRT; (**C**) ABSI and AFT; (**D**) ABSI and DSST

The relationships between ABSI and cognitive function are illustrated in Table 2. In the fully adjusted model (Model 3), a significant negative correlation was observed between ABSI and cognitive function. Specifically, an increase of one unit in ABSI was associated with a reduction in IRT scores by 52.46 points [β=-52.46, (-88.20, -16.72)], DRT by 17.72 points [β=-17.72, (-36.20, 0.76)], AFT scores by 23.96 points [β=-23.96, (-67.79, 19.87)], and DSST scores by 220.18 points [β=-220.18, (-326.96, -113.39)]. However, the negative associations between ABSI, DRT, and AFT were insignificant (Table 2). Comparisons between individuals in the highest and lowest tertiles of ABSI revealed declines in IRT, DRT, AFT, and DSST scores of 0.42, 0.05, 0.21, and 2.37, respectively. Smoothed curve fitting (Fig. 2) further substantiated the negative relationship between ABSI and cognitive function.

**Table 2:** Associations between ABSI and cognitive function

Cognitive function	Model1 β(95%CI) *P* value	Model2 β(95%CI) *P* value	Model3 β(95%CI) *P* value
IRT	-169.74 (-205.42, -134.06) < 0.0001	-75.37 (-111.25, -39.49) < 0.0001	-52.46 (-88.20, -16.72) 0.0040
T1	Ref	Ref	Ref
T2	-1.21 (-1.61, -0.81) < 0.0001	-0.62 (-1.01, -0.23) 0.0018	-0.50 (-0.89, -0.12) 0.0098
T3	-1.71 (-2.11, -1.31) < 0.0001	-0.66 (-1.06, -0.26) 0.0012	-0.42 (-0.81, -0.02) 0.0397
P for trend	< 0.0001	0.0014	0.0451
DRT	-72.66 (-90.91, -54.41) < 0.0001	-25.43 (-43.85, -7.01) 0.0068	-17.72 (-36.20, 0.76) 0.0604
T1	Ref	Ref	Ref
T2	-0.35 (-0.56, -0.14) 0.0010	-0.06 (-0.26, 0.14) 0.5658	-0.02 (-0.22, 0.18) 0.8353
T3	-0.68 (-0.89, -0.48) < 0.0001	-0.15 (-0.35, 0.05) 0.1488	-0.05 (-0.26, 0.15) 0.6144
P for trend	< 0.0001	0.1468	0.6129
AFT	-99.01 (-145.15, -52.86) < 0.0001	-60.05 (-105.50, -14.61) 0.0097	-23.96 (-67.79, 19.87) 0.2840
T1	Ref	Ref	Ref
T2	-0.46 (-0.98, 0.06) 0.0850	-0.35 (-0.85, 0.14) 0.1599	-0.13 (-0.60, 0.34) 0.5970
T3	-1.06 (-1.57, -0.54) < 0.0001	-0.60 (-1.10, -0.10) 0.0196	-0.21 (-0.69, 0.28) 0.4054
P for trend	< 0.0001	0.0200	0.4071
DSST	-695.95 (-828.17, -563.73) < 0.0001	-410.52 (-529.93, -291.10) < 0.0001	-220.18 (-326.96, -113.39) < 0.0001
T1	Ref	Ref	Ref
T2	-5.24 (-6.73, -3.75) < 0.0001	-3.44 (-4.74, -2.15) < 0.0001	-2.48 (-3.62, -1.34) < 0.0001
T3	-7.51 (-8.98, -6.04) < 0.0001	-4.28 (-5.61, -2.96) < 0.0001	-2.37 (-3.55, -1.19) < 0.0001
P for trend	< 0.0001	< 0.0001	0.0001

Model 1: variables were not adjusted. Model 2: adjustments were made to age, gender, and race. Model 3: Adjustments were made for age, gender, race, education level, PIR, HBP, diabetes, smoking status, and alcohol intake. *Abbreviations*: ABSI A Body Shape Index, IRT Immediate Recall test, DRT Delayed Recall test, AFT Animal Fluency Test, DSST Digit Symbol Substitution Test, PIR Poverty Income Ratio, HBP High Blood Pressure

The results of subgroup analyses and interaction tests show differential effects of ABSI on cognitive performance across various demographic segments, such as gender, age, race, smoking, alcohol intake, HBP, and diabetes (Table 3). Notably, males displayed a significantly more pronounced negative correlation between ABSI and DSST performance compared to females (*P* = 0.0025). Individuals consuming fewer than 12 alcoholic drinks annually showed a stronger negative association between ABSI and AFT scores compared to those who drank more (*P* = 0.002). Additionally, participants with HBP exhibited a significantly stronger negative relationship between ABSI and AFT scores compared to those without HBP (*P* = 0.0196). For other demographic categories, the associations between ABSI and cognitive function did not show statistical significance (*P* > 0.05).

**Table 3:** Subgroup analysis of the associations between ABSI and cognitive function

Subgroup	IRT recall β(95%CI)	*P* for interaction	DRT β(95%CI)	*P* for interaction	AFT β(95%CI)	*P* for interaction	DSST β(95%CI)	*P* for interaction
Age		0.5629		0.3741		0.9299		0.2276
<70	-62.44 (-111.15, -13.74)		-23.12 (-48.47, 2.23)		-42.80 (-102.29, 16.69)		-383.33 (-531.06, -235.60)	
≥ 70	-83.13 (-134.74, -31.52)		-39.67 (-66.53, -12.80)		-46.64 (-109.68, 16.40)		-252.44 (-408.98, -95.90)	
Gender		0.6417		0.9572		0.8067		0.0025
Male	-69.12 (-132.08, -6.16)		-18.94 (-51.41, 13.53)		-37.23 (-114.13, 39.66)		-452.77 (-639.63, -265.90)	
Female	-51.00 (-95.01, -6.99)		-20.02 (-42.71, 2.68)		-25.60 (-79.35, 28.15)		-103.20 (-233.81, 27.42)	
Race		0.8236		0.7073		0.7147		0.426
Mexican American	3.95 (-199.52, 207.42)		-12.09 (-117.14, 92.96)		-155.07 (-404.82, 94.68)		-251.09 (-857.65, 355.47)	
Other Hispanic	50.21 (-162.04, 262.45)		18.88 (-90.71, 128.46)		60.46 (-200.05, 320.98)		352.99 (-279.72, 985.70)	
Non-Hispanic White	59.46 (-99.37, -19.55)		-15.14 (-35.75, 5.46)		-21.47 (-70.46, 27.51)		-251.72 (-370.69, -132.75)	
Non-Hispanic Black	-91.36 (-217.87, 35.14)		-66.63 (-131.95, -1.32)		-38.33 (-193.61, 116.95)		-96.47 (-473.59, 280.65)	
Non-Hispanic Asian	7.58 (-216.68, 231.85)		-15.55 (-131.34, 100.23)		56.50 (-218.77, 331.76)		-5.88 (-674.41, 662.66)	
Other Race	37.14 (-384.61, 458.89)		32.78 (-184.97, 250.53)		240.42 (-277.25, 758.09)		257.33 (-999.91, 1514.57)	
Alcohol intakes ≥ 12drinks/year		0.4101		0.3777		0.002		0.0627
Yes	-44.08 (-87.13, -1.03)		-12.95 (-35.18, 9.27)		19.42 (-33.25, 72.09)		-149.51 (-277.94, -21.07)	
No	-76.81 (-142.31, -11.30)		-31.04 (-64.86, 2.77)		-130.53 (-210.67, -50.39)		-370.14 (-565.56, -174.72)	
Smoked ≥ 100 cigarettes		0.5346		0.8507		0.9281		0.5822
Yes	-65.79 (-119.37, -12.22)		-16.26 (-43.97, 11.46)		-23.72 (-89.45, 42.00)		-253.02 (-412.77, -93.27)	
No	-43.10 (-91.35, 5.16)		-19.81 (-44.78, 5.15)		-19.67 (-78.87, 39.52)		-193.05 (-336.94, -49.17)	
HBP		0.882		0.0641		0.0186		0.9212
Yes	-55.93 (-101.67, -10.19)		-31.81 (-55.49, -8.14)		-62.95 (-119.09, -6.81)		-228.22 (-365.09, -91.36)	
No	-50.39 (-108.19, 7.42)		3.98 (-25.94, 33.90)		45.01 (-25.93, 115.95)		-239.27 (-412.22, -66.32)	
Diabetes		0.2794		0.0652		0.6181		
Yes	-26.50 (-110.47, 57.47)		19.41 (-23.88, 62.70)		5.31 (-97.64, 108.26)		-56.13 (-306.26, 194.00)	0.2815
No	-70.42 (-111.68, -29.17)		31.06 (-52.33, -9.79)		-34.93 (-85.51, 15.66)		-251.09 (-373.99, -128.19)	
Broadline	42.87 (-112.23, 197.96)		23.92 (-56.05, 103.88)		41.43 (-148.73, 231.59)		-23.97 (-485.99, 438.04)	

Age, gender, race, education level, PIR, smoking, alcohol intake, HBP, and diabetes are adjusted

*Abbreviations* ABSI A Body Shape Index, IRT Immediate Recall test, DRT Delayed Recall test, AFT Animal Fluency Test, DSST Digit Symbol Substitution Test, PIR Poverty Income Ratio, HBP High Blood Pressure

### Comparative analysis of predictive models for cognitive impairment

**Fig. 3:**
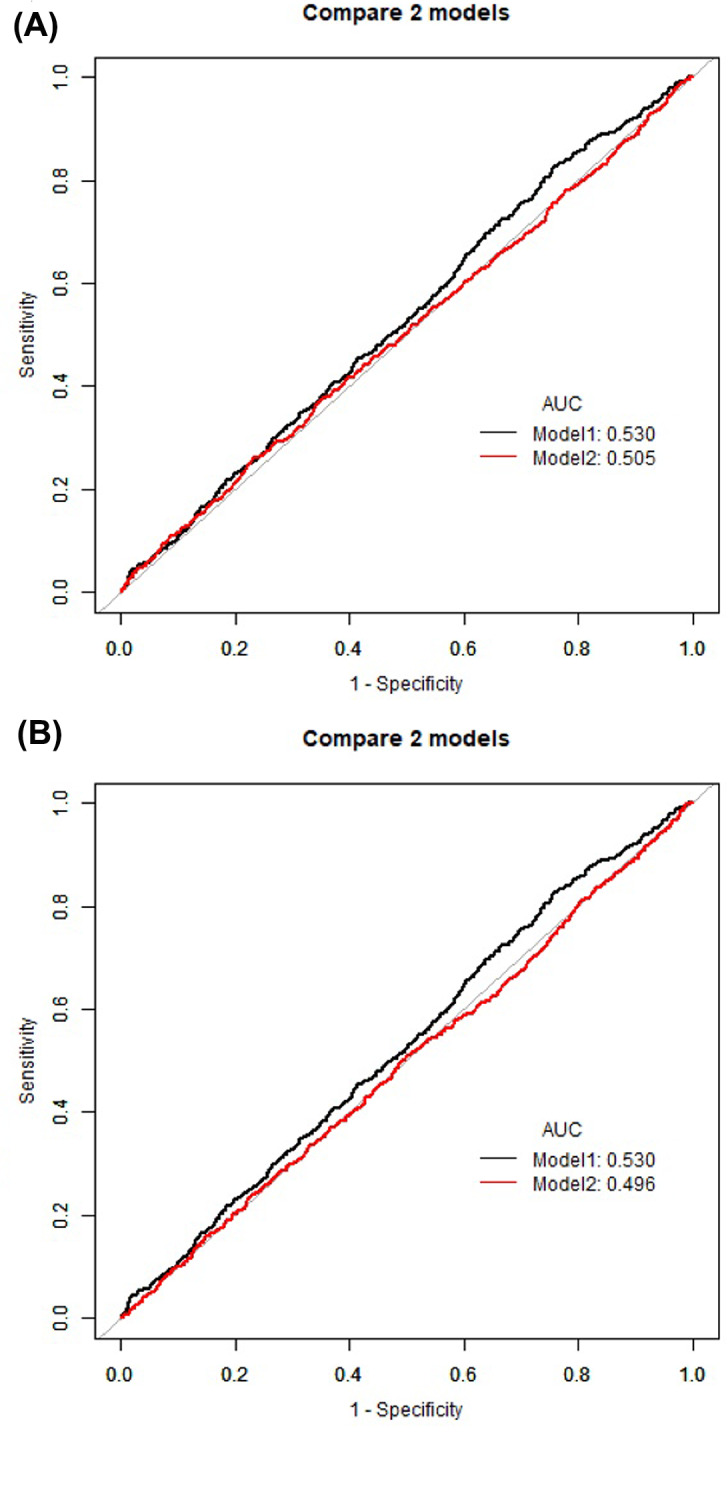
ROC curves for ABSI, BMI, and waist circumference. Legend for Figure 3A Description: This graph compares the discriminative ability of ABSI and BMI in predicting low cognitive function. Black Line (Model 1): Represents the ROC curve for ABSI as the sole predictor, with an AUC of 0.530. Red Line (Model 2): Represents the ROC curve for BMI as the sole predictor, with an AUC of 0.505. Legend for Figure 3B Description: This graph compares the discriminative ability of ABSI and WC in predicting low cognitive function. Black Line (Model 1): Represents the ROC curve for ABSI as the sole predictor, with an AUC of 0.530. Red Line (Model 2): Represents the ROC curve for WC as the sole predictor, with an AUC of 0.496

The ROC curve analyses conducted in this study explored the effectiveness of BMI, ABSI, and WC in predicting low cognitive function. In Fig. 3A, the ROC curve for ABSI (Model 1) displayed an AUC of 0.530, indicating moderate discriminative power. In contrast, the ROC curve for BMI (Model 2) showed a slightly lower AUC of 0.505, suggesting a marginally lesser discriminative capability. Similarly, in Fig. 3B, ABSI’s performance remained consistent with an AUC of 0.530, while WC, depicted in the second model, demonstrated an even lower AUC of 0.496. These results indicate that ABSI, while not substantially powerful, consistently outperformed both BMI and WC in predicting cognitive impairment.

## Discussion

In this study, 2752 older individuals in the US were evaluated to ascertain the association between cognitive impairment and ABSI. The results indicated that both DSST and IRT exhibited a significant negative correlation with ABSI in the fully adjusted model (*P* < 0.05). Consequently, memory loss and reduced processing speed are frequently observed in older individuals with higher ABSI. A trend test employing ABSI tertiles as an indicator also revealed a negative linear trend between ABSI and IRT, DSST scores, further substantiating their negative linear correlation. ABSI demonstrated a slightly higher predictive accuracy than BMI and WC. Additionally, while this study identified associations between ABSI, BMI, and WC with cognitive function, the ROCs indicate that none of these measures alone are strong predictors of cognitive function. Specifically, the AUROCs for ABSI, BMI, and WC were all below the threshold that would suggest good predictive power. This underscores the complexity of cognitive impairment and suggests that a combination of other factors alongside these anthropometric measures may provide a more accurate prediction of cognitive function.

This study explored the association between cognitive function and ABSI, identifying a link between declining cognitive function and increased ABSI. Indeed, utilizing BMI as a way of measuring obesity has limitations as BMI does not identify the distribution of body fat nor differentiate between muscle and fat tissue [15]. The primary limitation of WC is its ignorance of weight and height, which can lead to incorrect estimations of obesity prevalence among taller or shorter individuals [16]. The first proposal for ABSI, a metric involving waist circumference, height, and weight that is statistically independent of BMI, aimed to enhance the assessment of central obesity and was introduced in 2012. Research has recorded a powerful relationship between ABSI and the risks of diabetes, metabolic syndrome-related conditions, and cardiovascular disease (CVD). Some studies indicate a stronger correlation between ABSI and early mortality compared to that between waist circumference or BMI [17, 18]. Additionally, ABSI has been linked to diabetes mellitus, metabolic syndrome, HBP, and all-cause mortality [11, 19–23]. Earlier research demonstrated a negative correlation between ABSI and cognitive functioning in older Chinese [24], consistent with the findings of a significant Taiwanese study [25]. Given significant differences in genetic backgrounds, and cultural, dietary, and lifestyle factors across regions, findings from Asian populations may not fully apply to diverse populations in the United States. Moreover, variations in socioeconomic status and healthcare systems and their impact on cognitive health can differ significantly between regions. Therefore, conducting this research in the US is crucial to clarify the universality and reliability of ABSI across different demographic backgrounds. This research is the inaugural study to explore the association between ABSI and cognitive performance in Americans aged 60 and above. It seeks to determine if findings from Asian studies are applicable in a Western context and further elucidate the association between cognitive function and ABSI.

One systematic review identified a direct correlation between midlife obesity and cognitive decline [9]. The confluence of diabetes, obesity, and an aging population may significantly exacerbate cognitive impairment and increase the risk of dementia. In obese individuals, neuronal death and various morphological changes are often detected in the hippocampal regions. For instance, weight gain is typically associated with a reduction in hippocampal volume. Reductions in hippocampal volume disrupt synaptic communication, which is essential for memory and learning. Memory impairment results from disrupted long-term potentiation in the CA1 region and the dentate gyrus [26, 27]. Elevated BMI can also result in atrophy of the temporal lobe [28]. Moreover, increases in body weight were linked to decreased densities in the frontal gyrus, nucleus accumbens, central gyrus, and gray matter of the hindbrain [29].

The association between obesity and cognitive dysfunction involves multiple biological pathways. Preliminary research suggests that obesity may directly impact brain health and function via mechanisms such as pro-inflammatory responses, oxidative stress, insulin resistance, hormonal imbalances, and others. There is substantial evidence connecting pro-inflammatory changes in the hippocampus to obesity-related cognitive impairments in animal models. Numerous studies have shown a correlation between these pro-inflammatory changes and notable behavioral alterations [30–33]. Obesogenic diets elevate systemic inflammation and quickly trigger a pro-inflammatory state of the central nervous system [34]. An increase in BMI was related to increased expression of IL-10(the anti-inflammatory cytokine) and the critical tissue damage marker nitric oxide synthase two in human cadaveric frontal brain autopsies [35]. Evidence suggests that pro-inflammatory signaling from white adipose tissue contributes to central inflammation in obesity [36]. Additionally, TNF-α obstructs the clearance of triacylglycerol and inhibits insulin-stimulated de novo lipogenesis [37]. Individuals with obesity exhibit markedly reduced immunological tolerance, leading to excessive production of inflammatory mediators such as IL-6, TNF-α, IL-1β, and IFN-γ, alongside a significant decrease in anti-inflammatory cytokines like IL-4 and IL-10. Furthermore, mounting evidence suggests that gut microorganisms contribute to the central inflammatory state associated with obesity [38, 39]. Oxidative stress is a pathophysiological change linked to obesity. Chronic low-level inflammation, disruptions in the adipose microenvironment, and mitochondrial dysfunction are key factors contributing to oxidative stress in obese tissues [40]. In diabetic brain tissues, oxidative stress affects inflammation, insulin resistance, and lipid metabolism, potentially influencing both physiological and pathological processes. Patients with diabetes are more likely to experience cognitive dysfunction due to oxidative stress, which accelerates aging, tau protein deposition, and neuronal death. The hormone insulin regulates glucose homeostasis. Beyond its primary effects, insulin is linked to various aspects of brain function, including neural growth, regulation of glucose, eating behaviors, and cognitive processes. Moreover, insulin mediates synaptic neurotransmission and influences neuronal and glial metabolism via molecular signaling pathways in vivo. Brain insulin resistance affects immunological, metabolic, and synaptic functioning. It is defined by the brain cells’ incapacity to react to insulin as usual. Insulin resistance in the brain is linked to type 2 diabetes [41]. One of the main features of many metabolic diseases-type 2 diabetes- is linked to cognitive impairment is reduced insulin sensitivity [42]. Insulin resistance was termed a significant risk factor for the onset of Alzheimer’s disease neuropathology and neurodegeneration, given the crucial function that insulin plays in brain physiology [43]. Another alteration associated with obesity is an increase in plasma leptin levels. The hormone leptin, which regulates satiety, is produced by white adipocytes. Leptin resistance develops when receptors for leptin signaling are compromised in obesity. Consequently, impaired satiety perception in individuals with obesity leads to reduced energy expenditure and nutrient overconsumption [44]. Given its function in promoting the synthesis of pro-inflammatory factors during the acute phase of inflammation, leptin may operate as a modulator of immunological and neuroendocrine signals [45]. Collectively, these biological changes can damage neurons and disrupt neurotransmitter transmission, potentially leading to cognitive decline.

The results of the subgroup analysis and interaction tests indicate that gender, alcohol consumption, and HBP status may modify the impacts of ABSI on cognitive functioning. This suggests that the association between ABSI and cognitive decline varies among individuals with different genders, drinking habits, and blood pressure levels. Studies have demonstrated that females perform more efficiently than males on the DSST [46]. Furthermore, females outperform males on the Letter Digit Substitution Test, which was derived from the DSST [47]. When compared to males, healthy older women have better verbal fluency, processing speed, and verbal memory [48]. These differences in cognitive abilities may be attributable to sex-based differences in brain function. The volume of grey matter in the posterior cerebellum, per temporal region, and bilateral orbitofrontal gyrus was significantly reduced in obese male subjects, whereas these changes were not evident in obese female samples [49]. Brain volume changes are primarily attributed to insulin resistance common in obesity, which hinders insulin transport to the brain, impairs glucose utilization by neuronal cells, and ultimately results in abnormalities in frontal axon and myelin structures, leading to cerebral atrophy [50]. Furthermore, it has been shown that the neuroprotective qualities of estrogen lower women’s risk of developing Parkinson’s disease [51]. Evidence suggests that estrogen positively affects the maintenance of neurocognitive integrity [52–54]. Variations in cognitive function between males and females may also stem from differences in dopamine concentrations. Female Parkinson’s patients exhibit higher dopamine concentrations than their male counterparts, which generally results in milder clinical symptoms [55–57].

The negative correlation between ABSI and AFT scores was no longer significant for the entire sample in the fully adjusted model (Model 3). Within particular subgroups (drinking and HBP subgroups), the interaction tests did, however, show a significant difference in the negative correlation between ABSI and AFT. This variation suggests that the impact of ABSI on AFT scores may depend on two factors: an individual’s drinking status and HBP presence. This fluctuation would suggest that, in these particular subgroups, there are other characteristics that modulate the association between ABSI and cognitive functioning, factors that are not as apparent in the general population. Individuals consuming 12 or more drinks annually exhibited a weaker negative correlation between ABSI and AFT compared to those who drank less. This may be attributed to the study’s criterion of a minimum of 12 drinks per year, suggesting that most older adults likely engage in mild to moderate consumption of alcohol. Individuals who drank alcohol from low to moderate levels showed lower rates of cognitive deterioration in all evaluated cognitive areas when compared to non-drinkers, indicating a protective relationship between mild to moderate alcohol intake and cognitive performance [58–60]. Researchers suggest that for all subjects, the optimal amount of alcohol consumption for improved cognitive performance is between 10 and 14 drinks per week [58]. It is yet unknown what processes underlie the link between mild to moderate alcohol use and improved cognitive function. The main hypotheses center on the functions of cerebral and cardiovascular networks, as well as brain-derived neurotrophic factors [61–65]. Alcohol may have a mixed influence on cognitive function since it affects the circulatory system in both positive and negative ways. For people who take mild to moderate amounts of alcohol, the positive effects on cardiovascular health may exceed the negative implications. Although high levels of alcohol intake do not change mRNA expression, moderate alcohol consumption increases levels of neurotrophic factors in the dorsal striatum, a significant regulative factor of neuronal plasticity and development [62]. Compared to subjects without HBP, those with HBP showed a more powerful negative association between ABSI and AFT. ABSI may be more strongly related to cognitive decline in individuals with HBP, suggesting that managing ABSI, such as reducing abdominal obesity, could effectively prevent or slow cognitive decline in this group. It is widely recognized that middle-aged HBP increases the risk of dementia [66]. HBP is significantly linked to cognitive decline and poor cognitive performance [67]. HBP is a prevalent disease among older adults, and it is recognized as a potential indicator of dementia. The integrity of the cerebrovascular system is continuously compromised by chronic hypertension. This can result in microvascular rarefaction, malfunction, and neurovascular uncoupling, all of which can have a negative impact on the cerebral blood supply. HBP induces neuroinflammation, damages the blood-brain barrier, and heightens the risk of amyloid buildup and Alzheimer’s disease [68]. These results indicate potential directions for future research. In populations with varying lifestyle habits (such as frequent alcohol intake) and health conditions (like HBP), the impact of ABSI on cognitive function may operate through specific physiological mechanisms related to body fat distribution, metabolic health status, or chronic disease effects. Gender interaction results may reveal differences in adiposity distribution, hormone levels, and social roles and behaviors between men and women, potentially influencing the pathways linking ABSI to cognitive function.

## Study strengths and limitations

This study is the first in the US to explore the link between cognitive performance and ABSI in individuals over 60, providing a new angle for future research to better clarify the connection between obesity and cognitive impairment. This study utilized a dataset from NHANES, including 2,752 older adults, enhancing the reliability and generalizability of the findings. The association between ABSI and scores on various cognitive function tests was thoroughly analyzed using multiple statistical techniques, contributing to the study’s rigor. According to the study’s findings, ABSI may significantly predict cognitive dysfunction in obese older adults, potentially aiding in early intervention.

This study does have certain limitations. Firstly, as a cross-sectional survey, it cannot build a causal relationship between ABSI and cognitive impairment. While the study identified an association between ABSI and poorer cognitive performance, this relationship may have been influenced by unconsidered confounding factors. Secondly, as this study focused on an older population in the US, its findings may not be found among younger or middle-aged adults. Additionally, while ABSI is a novel index for assessing abdominal obesity, further research is required to validate its superiority over traditional metrics such as BMI or WC. This study was not able to find any significant associations between ABSI and DRT scores, potentially due to the limited sensitivity of ABSI and DRT, and the heterogeneity in the sample’s health status and lifestyle factors. Future research may benefit from using more sensitive measures and more prominent, more diverse samples to figure out the complex relationships between obesity indices and cognitive functioning. Lastly, this study did not investigate the various mechanisms underlying the link between ABSI and cognitive impairment.

## Conclusion

This study identified a significant negative correlation between ABSI and cognitive function in older adults in the US. Higher ABSI was linked to lower scores on cognitive tests, especially the IRT and DSST. These results suggest that central obesity could be a risk factor for cognitive decline.

The clinical significance of our findings implies that assessing ABSI in older adults may help identify those at higher risk for cognitive impairment. This knowledge can inform tailored interventions aimed at reducing central obesity, such as dietary modifications, physical activity programs, and other lifestyle changes, potentially mitigating cognitive decline. Incorporating ABSI into routine clinical assessments might enhance the early detection of cognitive risks and guide personalized care strategies.

Future research should prioritize longitudinal studies to validate these findings and investigate the underlying biological mechanisms. Furthermore, investigating how specific therapies affect ABSI and cognitive function is essential to creating preventative and therapeutic plans that effectively address the cognitive deterioration linked to central adiposity.

## Data Availability

The datasets collected and examined in this research can be found at [https://www.cdc.gov/nchs/nhanes/index.htm], the NHANES repository.

## Abbreviations

ABSI: A Body Shape Index
IRT: Immediate Recall test
DRT: Delayed Recall test
AFT: Animal Fluency Test
DSST: Digit Symbol Substitution Test
WC: Waist Circumference
NHANES: National Health and Nutrition Examination Survey
BMI: Body Mass Index
MCI: Mild Cognitive Impairment
CVD: Cardiovascular disease
PIR: Poverty Income Ratio
HBP: High Blood Pressure
CERAD W-L: Consortium to Establish a Registry for Alzheimer’s Disease Word Learning
ROC: Receiver Operating Characteristic
AUC: Area Under the Curve
