# Correction: Association between a body shape index and cognitive impairment among US older adults from a cross-sectional survey of the NHANES 2011–2014

**DOI:** 10.1186/s12944-024-02187-w

**Published:** 2024-06-26

**Authors:** Yanwei Zhang, Peng Zhang, Dekun Yin

**Affiliations:** 1 Department of Anesthesiology, Shanghai Ninth People’s Hospital, Shanghai Jiao Tong University School of Medicine, Shanghai, China; 2 Department of Anesthesiology, Funing People’s Hospital of Jiangsu, Yancheng, Jiangsu province China

**Correction: Lipids Health Dis 23, 169 (2024)**

10.1186/s12944-024-02165-2

Following publication of the original article [1], the Editors of the journal reported a typo to the article title. The typo is presented in bold below.

The incorrect article title is: Association between a body shape index and cognitive impairment among **us** older adults from a cross-sectional survey of the NHANES 2011–2014

The correct article title is: Association between a body shape index and cognitive impairment among **US** older adults from a cross-sectional survey of the NHANES 2011–2014

The original article [1] has been updated.
